# Review of the Book Principles and Practice of Particle Therapy, Edited by Timothy D. Malouff and Daniel M. Trifiletti, 1st Edition 111 River Street, Hoboken, NJ 07030, USA: John Wiley & Sons Ltd, 2022 p.560. ISBN: 9781119707516

**DOI:** 10.1002/jmrs.763

**Published:** 2024-02-23

**Authors:** Milad Mirzaei

**Affiliations:** ^1^ Radiation Oncology Sir Charles Gairdner Hospital, Hospital Avenue Nedlands Western Australia Australia

## Abstract

The *Principles and Practice of Particle Therapy* methodically compiles important concepts in particle therapy (PT) such as medical physics, radiobiology, treatment planning, image guidance, treatment delivery, advanced technologies, clinical indications and considerations for various anatomical disease sites. Undoubtably, this book is a clinically oriented resource providing a practical guide for radiation oncologists, medical physicists, radiation therapists and students who wish to learn about PT.

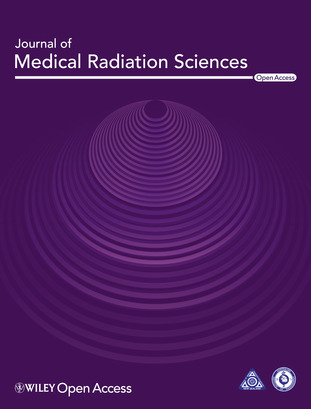

Particle therapy (PT) has revolutionised cancer treatment, and the number of centres offering such advanced technology is rapidly growing around the world.

The *Principles and Practice of Particle Therapy* methodically compiles important concepts in PT such as medical physics, radiobiology, treatment planning, image guidance, treatment delivery, advanced technologies, clinical indications and considerations for various anatomical disease sites. Undoubtably, this book is a clinically oriented resource providing a practical guide for radiation oncologists, medical physicists, radiation therapists and students who wish to learn about PT. This book was published in May 2022 by John Wiley & Sons Ltd. and written by world leading experts with broad clinical experience in radiation oncology and PT physics.

This book is divided into three sections covering a total of 27 chapters.

**Chapter 1:**

This chapter briefly describes the historical evolution of PT and introduces the key pioneers at a few institutions that developed highly sophisticated modalities for targeting cancer.

**Chapter 2:**

This chapter provides readers with background information about the physics of conventional radiation therapy (RT). Also, it describes various types of therapeutic and non‐therapeutic neutrons as well as their interaction with matter. The physics of helium, carbon ion and protons as well as the production of subatomic particles are discussed in this chapter.

**Chapter 3:**

Radiobiological characteristics of PT are described in this chapter. The effects of high and low linear energy transfer radiation on human cells are highlighted including different types of cellular damage. Topics such as relative biological effectiveness, bystander effect, cell signalling and experimental techniques in particle‐induced cellular damage are thoroughly covered.

**Chapter 4:**

This chapter provides an overview of three powerful accelerators (i.e., cyclotrons, synchrotron and synchrocyclotron) that are currently available for proton beam therapy (PBT). The schematics and equations enable readers to better understand the physics of accelerators and their principle of operations.

**Chapter 5:**

The focus of this chapter is the principles of treatment planning for scanning PBT. Various exercises, figures and explanations are outlined to educate readers about the dosimetric differences between proton and photon therapies. Important concepts such as planning margins, uncertainties, optimisation techniques (e.g., single‐ and multi‐field optimisation) and planning challenges are discussed. Furthermore, this chapter demonstrates the potential of a novel treatment planning technique known as ‘individual field simultaneous optimisation’ as well as other robust approaches for head and neck (HN), breast, gynaecological and anorectal tumours. Most importantly, it provides planning strategies and a specific list of Do's and Don'ts for treatment planners; however, they are mostly derived from the observations and studies conducted at a single institution.

**Chapter 6:**

This chapter focusses on immobilisation, image acquisition and guidance, motion management and challenges involving dose calculation algorithms in PBT. Also, it deals with strategies for managing image artefacts and heterogeneities, which can result in discrepancies between planned and delivered doses. Furthermore, this chapter outlines workflows for robust treatment planning and optimisation in the presence of moving targets and the interplay effect. Moreover, highly advanced imaging technologies such as prompt gamma and proton computed tomography are discussed.

**Chapter 7–10:**

Chapter 7 discusses advanced modalities for PT delivery such as intensity‐modulated proton therapy (IMPT), spot scanning proton arc therapy and other future modalities for PT (e.g., multi‐ion PT). Chapter 8 covers proton and electron FLASH radiotherapy. The results of various in vivo and in vitro studies are presented in this chapter. Also, the technical challenges in developing FLASH programs are discussed. Chapter 9 thoroughly discusses boron neuron capture therapy, boron pharmacokinetics, neutron sources, treatment planning and clinical applications for different disease sites. A summary of grid therapy (e.g., microbeam and minibeam radiotherapy) and its radiobiological principles is outlined in chapter 10.

**Chapter 11:**

One aspect of increasing importance in radiation oncology is the immune response to ionising radiation. Chapter 11 focusses on cancer immunoediting, immunotherapy pathways and how PT enhances the immunogenicity of cells. In addition, various in vivo and in vitro studies are highlighted.

**Chapter 12:**

This chapter reviews the economics of neutron and carbon ion therapies but mainly focusses on PBT and its cost effectiveness in the United States. In addition, it provides a list of PBT centres per country and the year they became clinically operational.

**Chapter 13–27:**

The third section of the book is categorised by disease site, and each chapter aims to provide clinical indications, treatment planning considerations, outcomes and future directions for health professionals.

The intracranial tumours are covered in Chapter 13. Passive scattering and uniform scanning techniques are briefly discussed. General treatment planning considerations are mainly for pencil beam scanning. Chapter 14 is the most comprehensive chapter covering ocular malignancies. The highlight of this chapter is the clinical experience, practical considerations, treatment planning and delivery systems that are covered in depth. Chapter 15 focusses on brain, skull base and spinal tumours. Some of the content of this chapter (e.g., meningioma and glioma) appears similar to Chapter 13 on intracranial tumours; however, studies on carbon ion treatments are explored in more detail. The section on treatment planning considerations is quite short and only highlights recommended target volumes and doses. Various types of HN malignancies are covered in Chapter 16. The main focus of this chapter is the ongoing clinical trials concerning PT for this patient group and considerations for different tumours. Chapter 17 discusses thoracic malignancies evaluating retrospective and prospective data to date. Chapter 18 compares the potential of PT modalities for treating different gastrointestinal tumours. Chapter 19 is a comprehensive review of hepatobiliary malignancies. The authors have compiled data from more than 300 references and have organised them into several tables. Male and female breast cancers are covered in Chapter 20. This chapter explores various PBT techniques and provides important treatment planning considerations.

Chapters 21 to 23 discuss prostate, non‐prostate genitourinary and gynaecological cancers in great depth, further exploring current and future PT modalities for eradicating tumours. Proton beam therapy for leukaemias and lymphomas of the HN, mediastinum, infradiaphragm and craniospinal irradiation are covered in Chapter 24. Chapter 25 outlines strategies for managing radioresistant or unresectable sarcomas and soft tissue cancers. Remarkably, this chapter also explores the applications of less commonly used PT modalities such as helium, neon and pions. Finally, the text concludes with Chapter 26 and 27 in which PT for paediatric tumours is extensively discussed. These two chapters highlight considerations for specific tumour types, also discuss several cases that were treated with proton therapy.

In summary, this is a well‐organised book that provides up‐to‐date information on PT obtained from high‐quality sources. The authors of the book have shared their tremendous knowledge and experience to broaden our views on PT, also to pave the way for future research in this field.

## Data Availability

Data sharing not applicable ‐ no new data generated, or the article describes entirely theoretical research.

